# Impact of integrated nursing intervention combined with psychological care on postoperative quality of life, negative emotions, and nursing satisfaction in patients with thyroid cancer

**DOI:** 10.3389/fmed.2025.1689245

**Published:** 2025-12-08

**Authors:** Meijuan Guo, Ping Huang

**Affiliations:** 1 Thyroid and Breast Surgery, Ganzhou People’s Hospital, Ganzhou, Jiangxi, China; 2 Department of vascular surgery, Ganzhou people’s Hospital, Ganzhou, Jiangxi, China

**Keywords:** comprehensive nursing intervention, psychological care, thyroid cancer, postoperative quality of life, negative emotions, nursing satisfaction

## Abstract

**Background:**

Thyroid cancer is a common endocrine system disorder, with surgery being the primary treatment. However, postoperative patients often face issues such as reduced quality of life, negative emotions, and unmet nursing needs. Traditional nursing models struggle to comprehensively address patients’ physical and psychological needs, making the exploration of more effective nursing approaches clinically significant.

**Objective:**

To investigate the impact of integrated nursing intervention combined with psychological care on postoperative rehabilitation outcomes in patients with thyroid cancer.

**Methods:**

This study employed a retrospective observational design. A total of 100 patients who underwent thyroid cancer surgery between January 2023 and June 2024 were retrospectively included, and divided into two groups according to the nursing intervention received: a control group (conventional nursing) and an observation group (integrated nursing combined with psychological care). The Anxiety Self-Rating Scale (SAS), Depression Self-Rating Scale (SDS), World Health Organization Quality of Life Brief Assessment Form (WHOQOL-BREF) scores, and nursing satisfaction were compared between the two groups before and after the intervention.

**Results:**

Following the intervention, the observation group exhibited significantly lower SAS and SDS scores compared to the control group (*P* < 0.05), as well as significantly higher WHOQOL-BREF scores and nursing satisfaction than the control group (*P* < 0.05).

**Conclusion:**

Comprehensive nursing combined with psychological care can effectively alleviate postoperative negative emotions in thyroid cancer patients, improve quality of life, and enhance nursing satisfaction.

## Introduction

Thyroid cancer, a common malignant tumor in the head and neck region, has seen a significant increase in global incidence in recent years. In urban women in China, it has risen to the fourth most common malignant tumor, with a continued annual growth rate of 20% (1, 2). Surgery is the primary treatment method for thyroid cancer. However, patients often face psychological issues such as anxiety and depression, as well as challenges like sleep disorders, fatigue, and reduced quality of life post-surgery (3, 4). Studies show that approximately 40% of thyroid cancer patients experience moderate to severe anxiety post-surgery, which directly impacts treatment adherence and recovery progress (5, 6).

Traditional conventional care models have limitations in addressing these complex issues (7). In recent years, integrated care, combined with psychological care, has emerged as a multidimensional intervention model that integrates physiological, psychological, and social support, demonstrating significant advantages in the management of cancer patients. A study by the First Affiliated Hospital of Fujian Medical University found that collaborative care, combined with mindfulness-based stress reduction, significantly improved patients’ emotional state and sleep quality (8). Effective, comprehensive care enhanced patients’ treatment adherence and satisfaction (9).

This study aims to thoroughly analyze the specific role of comprehensive nursing intervention combined with psychological care in the postoperative recovery process of thyroid cancer patients, and to accurately assess its impact on patients’ quality of life, negative emotions, and nursing satisfaction. By retrospectively analyzing the clinical data of 100 thyroid cancer patients treated at our hospital, the study compares the differences in postoperative quality of life, negative emotions, and nursing satisfaction between those who received routine care and those who received comprehensive nursing intervention combined with psychological care (10, 11). This study aims to provide a scientific and robust basis for clinical nursing practices, assist in optimizing nursing plans, and further improve the quality of care for thyroid cancer patients, thereby promoting better recovery outcomes and effectively enhancing their prognosis and quality of life.

This study targeted thyroid cancer patients who underwent surgery between 2023 and 2024, aiming to systematically analyze the intervention effects of comprehensive care combined with psychological care on patients postoperative negative emotions, quality of life, and nursing satisfaction, providing evidence-based guidance for clinical practice.

## Materials and methods

### Study population

A total of 100 patients undergoing surgery for thyroid cancer admitted between January 2023 and June 2024 were retrospectively selected and divided into two groups according to the nursing intervention they received: the observation group (*n* = 50, 22 males and 28 females; age range 32–58 years, mean age 45.3 ± 8.7 years; pathological types included 38 cases of papillary carcinoma and 12 cases of follicular carcinoma) and the control group (*n* = 50, 24 males and 26 females; age range 31–60 years, mean age 46.1 ± 9.2 years; 36 cases of papillary carcinoma and 14 cases of follicular carcinoma). Inclusion criteria: ➀ pathologically confirmed differentiated thyroid carcinoma; ➁ scheduled for total thyroidectomy; ➂ normal cognitive function able to cooperate with questionnaire surveys; ➃ provided verbal or documented clinical informed consent. Exclusion criteria: ➀ comorbid severe cardiac, hepatic, or renal diseases; ➁ history of psychiatric illness; ➂ distant metastasis or advanced-stage disease. Baseline characteristics showed no statistically significant differences between the two groups (*P* > 0.05), indicating comparability (shown in Table 1). This study was approved by the hospital’s ethics committee (Approval No. IRB-2022-089).

**TABLE 1:** Comparison of baseline data between the two groups of patients.

Project	Observation group (*n* = 50)	Control group (*n* = 50)	*P*
Age (years)	45.3 ± 8.7	46.1 ± 9.2	> 0.05
Gender (male/female)	22/28	24/26	> 0.05
Pathological type			> 0.05
Papillary carcinoma	38	36	
Follicular carcinoma	12	14	
Type of tumor			>0.05
Benign	30	29	
Malignant	20	21	
Surgical approach			>0.05
Partial resection	19	21	
Total resection	31	29	

### Intervention methods

#### Control group intervention

The control group was given routine perioperative care for thyroid cancer, including: Preoperative preparation: Complete all preoperative examinations, including blood tests, coagulation function tests, liver and kidney function tests, thyroid function tests, neck ultrasound, and electrocardiograms, to assess the patients’ physical condition comprehensively, determine the surgical indications, and evaluate the patients’ tolerance for surgery. Provide detailed information to the patient and their family about the purpose of the surgery, the general procedure, potential risks, and the postoperative recovery process, to ensure they have a thorough understanding of the surgery and to alleviate their fears and concerns. Guide the patient in performing respiratory exercises, such as deep breathing and effective coughing, to prevent postoperative lung complications. Train the patient to adapt to the surgical position, which involves placing pillows under the shoulders and back, lying flat with the head tilted back, and exposing the neck as much as possible. Practice this several times a day, gradually increasing the duration of each session, to enhance the patient’s tolerance to the surgical position and avoid discomfort during surgery. Prepare the skin by cleaning the neck, and if a neck dissection is suspected due to a malignant tumor, shave the hair behind both ears (12).

During the operation, assist in positioning the patient to ensure their comfort and safety. Closely monitor vital signs, including heart rate, blood pressure, breathing, and oxygen saturation, and report any abnormalities to the surgeon promptly. Strictly adhere to aseptic techniques, work closely with the surgeon to complete the surgical procedures, and accurately transfer surgical instruments and items to ensure the smooth progress of the surgery.

#### Postoperative basic care

Closely monitor the patient’s vital signs, including breathing, pulse, blood pressure, and body temperature, every 30 min to 1 h until the vital signs stabilize. Once the patient is conscious and their blood pressure is stable, please place them in a semi-recumbent position to facilitate breathing and coughing up phlegm, ensuring the airway remains clear. This also facilitates wound drainage, thereby reducing the risk of fluid accumulation and infection. Keep the wound dressing clean and dry, and replace any soaked dressings promptly (3). Watch for any signs of bleeding, exudate, redness, swelling, or pain at the wound site. If significant bleeding occurs, notify the doctor immediately. Secure the drainage tube properly to ensure it remains unobstructed, avoiding twisting, compression, or blockage. Monitor the amount, color, and nature of the drainage fluid. If the drainage fluid is excessive, bright red, or abnormally turbid, it may indicate complications such as bleeding or infection and should be reported to the doctor immediately (13). If there are no symptoms, such as nausea or vomiting, 6 h after surgery, the patient can be given a small amount of warm water to drink. Over the next 1–2 days, gradually transition the diet to a liquid or semi-liquid state, and then to a regular diet. The diet should be light and easy to digest, rich in protein, vitamins, and minerals, and avoid spicy and irritating foods to minimize irritation to the wound.

#### Observation group intervention

The observation group received integrated nursing care combined with psychological interventions for 3 months in addition to routine care. including: ➀ Weekly family conferences were held to develop personalized rehabilitation plans. During these conferences, family members’ roles in pain monitoring and medication supervision were clearly defined. ➁ Mindfulness-based interventions: 30-min mindfulness breathing and body scan exercises were conducted twice weekly to promote emotional acceptance (14). Additionally, positive psychological reconstruction was enhanced by sharing success stories (e.g., video interviews of patients who survived 10 years after surgery (14)). ➂ Progressive muscle relaxation: Implemented from postoperative day 3 (15 min twice daily), systematically relaxing hand, shoulder/neck (key area), and lower limb muscle groups to reduce surgical site tension. ➃ WeChat-based transitional care: Daily rehabilitation videos (e.g., neck function exercises) were shared via group chat. Expert Q&A sessions were held every Wednesday at 7:00 p.m. Meanwhile, real-time data were collected through the Questionnaire Star mini-program. These data were used to dynamically adjust pain and sleep interventions (15). ➄ Cognitive Behavioral Therapy (CBT): Administered by psychiatric nurses to patients with anxiety/depression scores > 50. Phase 1 (week 1): Identifying automatic negative thoughts (e.g., “cancer will inevitably recur”); Phase 2 (weeks 2–4): Behavioral activation through daily pleasure scheduling; Phase 3 (weeks 5–12): Cognitive restructuring of catastrophic thinking.

### Observation indicators

#### Negative emotion assessment

Before and 3 months after the intervention, the negative emotions of patients in both groups were assessed using the Self-Rating Anxiety Scale (SAS) and the Self-Rating Depression Scale (SDS). These scales are widely used in clinical settings for their high reliability and validity, effectively measuring anxiety and depression levels (16). The SAS consists of 20 items, assessing the frequency of symptoms as defined by the items, using a 4-point rating scale: “1” indicates no or rarely experiencing symptoms; “2” indicates occasionally experiencing symptoms; “3” indicates frequently experiencing symptoms; “4” indicates almost always or always experiencing symptoms. Items 5, 9, 13, 17, and 19 are scored in reverse order. The total score is obtained by summing the scores of all 20 items, then multiplying by 1.25 to round up to the nearest whole number, which is the standard score. A standard score below 50 indicates normal; 50–60 indicates mild anxiety; 61–70 indicates moderate anxiety; and above 70 indicates severe anxiety. The SDS also consists of 20 items, using a 4-point rating scale from “none” to “persistent,” with scores ranging from 1 to 4. Ten items are scored in reverse. The total score is obtained by summing the scores of all 20 items and then converting it into a standard score. A standard score below 53 indicates normal; 53–62 indicates mild depression. Those with scores between 63 and 72 are classified as having moderate depression, while those with scores above 72 are classified as having severe depression. By assessing patients’ SAS and SDS before and after the intervention, we can accurately understand the changes in their negative emotions, providing quantitative data to evaluate the improvement effect of the comprehensive nursing intervention combined with psychological nursing on patients’ mental state.

#### Changes in quality of life

The WHOQOL-BREF comprises 26 self-report items (24 domain-specific + 2 global) rated on a 5-point Likert scale (e.g., “Not at all” to “Completely”) over the past 2 weeks, with four domains: Physical Health (7 items covering energy, pain, sleep, daily activities), Psychological Health (6 items including positive feelings, self-esteem, spirituality), Social Relationships (3 items involving satisfaction with personal relationships and social support), and Environment (8 items such as safety, living conditions, healthcare access); in terms of scoring, raw scores are transformed into standardized scores (0–100) where higher values indicate better quality of life, with domain scores calculated by averaging item scores and multiplying by 4 (to align with WHOQOL-100)—for example, a Physical Health score of 70 suggests above-average physical wellbeing.

#### Nursing satisfaction assessment

Before the patient is discharged, the nursing satisfaction of patients in two groups was assessed using a questionnaire. The hospital-designed questionnaire, which underwent multiple rounds of expert review and pre-survey, demonstrated good reliability and validity. The questionnaire covered 15 items, including nursing service attitude, nursing technical skills, health education effectiveness, ward environment comfort, and the timeliness of responding to patient needs. Each item was rated on a 5-point Likert scale, ranging from “very satisfied” to “very dissatisfied,” with scores ranging from 5 to 1. The total score was calculated by summing the scores of all items. A total score of 60 or higher indicates very satisfied; 45–59 indicates satisfied; 30–44 indicates average; and less than 30 indicates dissatisfied. Satisfaction was calculated as (number of very satisfied + number of satisfied)/total number of cases × 100%. The questionnaire method enables the direct collection of patients’ subjective evaluations of nursing work, thereby understanding their needs and expectations, and provides a reference for improving nursing work and enhancing nursing quality.

### Statistical analysis

Data were analyzed using SPSS 17.0 statistical software. Quantitative data are expressed as (x ± s). Intergroup and intragroup comparisons were performed using *t*-tests, while intergroup comparisons of count data were performed using χ^2^-tests. *P* < 0.05 was considered statistically significant.

## Results

### Improvement in negative emotions

**FIGURE 1:**
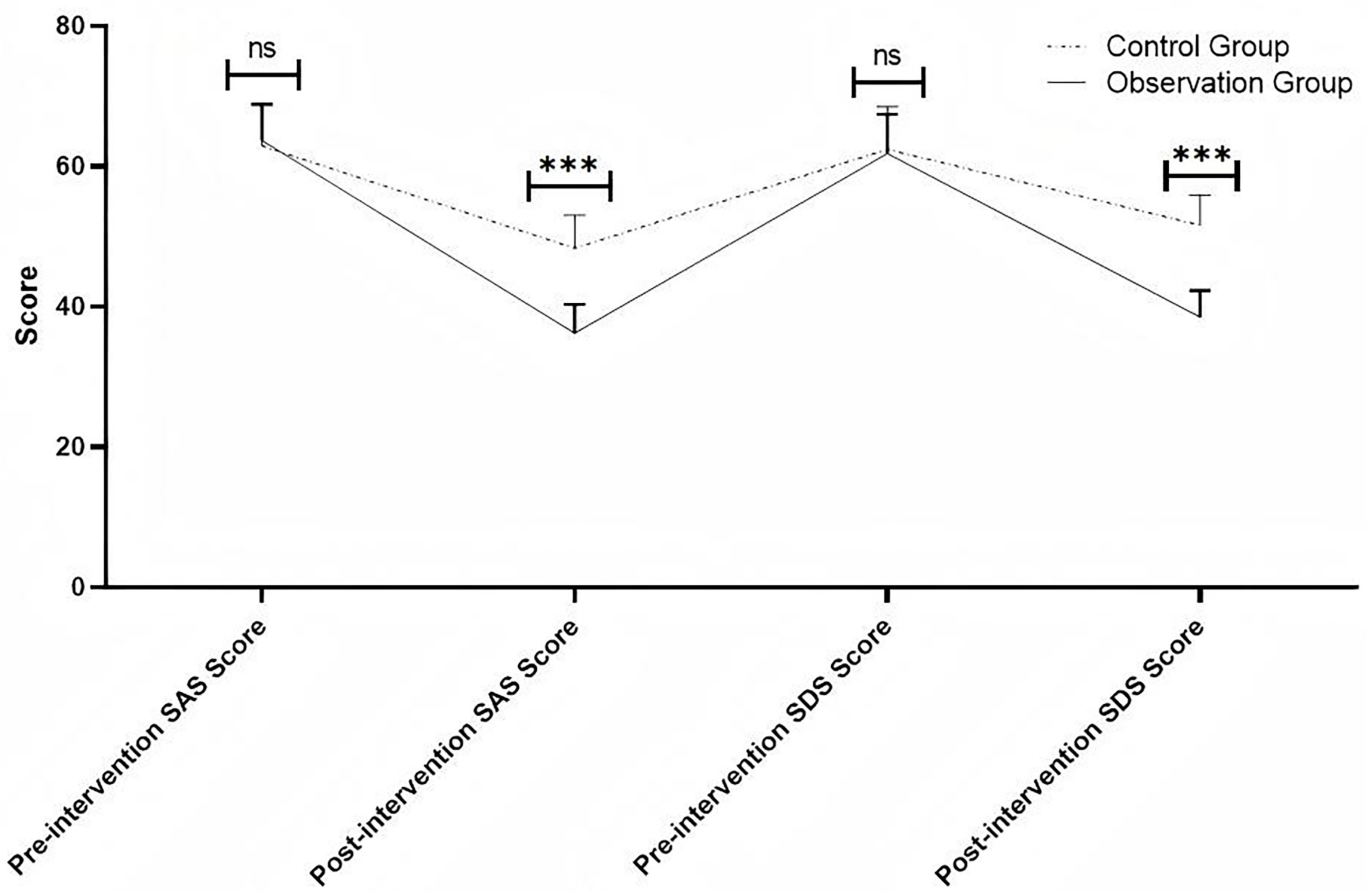
Comparison of psychological status scores (SAS and SDS) between the two groups before and after intervention. ***P < 0.001.

There was no significant difference in SAS and SDS scores between the two groups before intervention (*P* > 0.05). After 3 months of intervention, the SAS and SDS scores in the observation group were significantly lower than those in the control group (*P* < 0.05) (Figure 1 and Table 2). Figure 1 intuitively displays the decreasing trend of anxiety and depression scores in both groups after intervention, with the observation group showing a more pronounced downward slope, further verifying the statistical difference between the two groups.

**TABLE 2:** Comparison of psychological status scores between the two groups before and after nursing (x̄ ± s, points).

Group	n	SAS score		SDS score	
		Pre-intervention	Post-intervention	Pre-intervention	Post-intervention
Observation group	50	63.7 ± 5.2	36.2 ± 4.1*	61.8 ± 5.6	38.5 ± 3.8*
Control group	50	62.9 ± 5.8	48.3 ± 4.7	62.4 ± 6.1	51.6 ± 4.3
*T*		0.72	13.85	0.53	16.22
*P*		> 0.05	< 0.001	> 0.05	< 0.001

Compared with the control group,

*P < 0.05.

### Changes in quality of life

**FIGURE 2:**
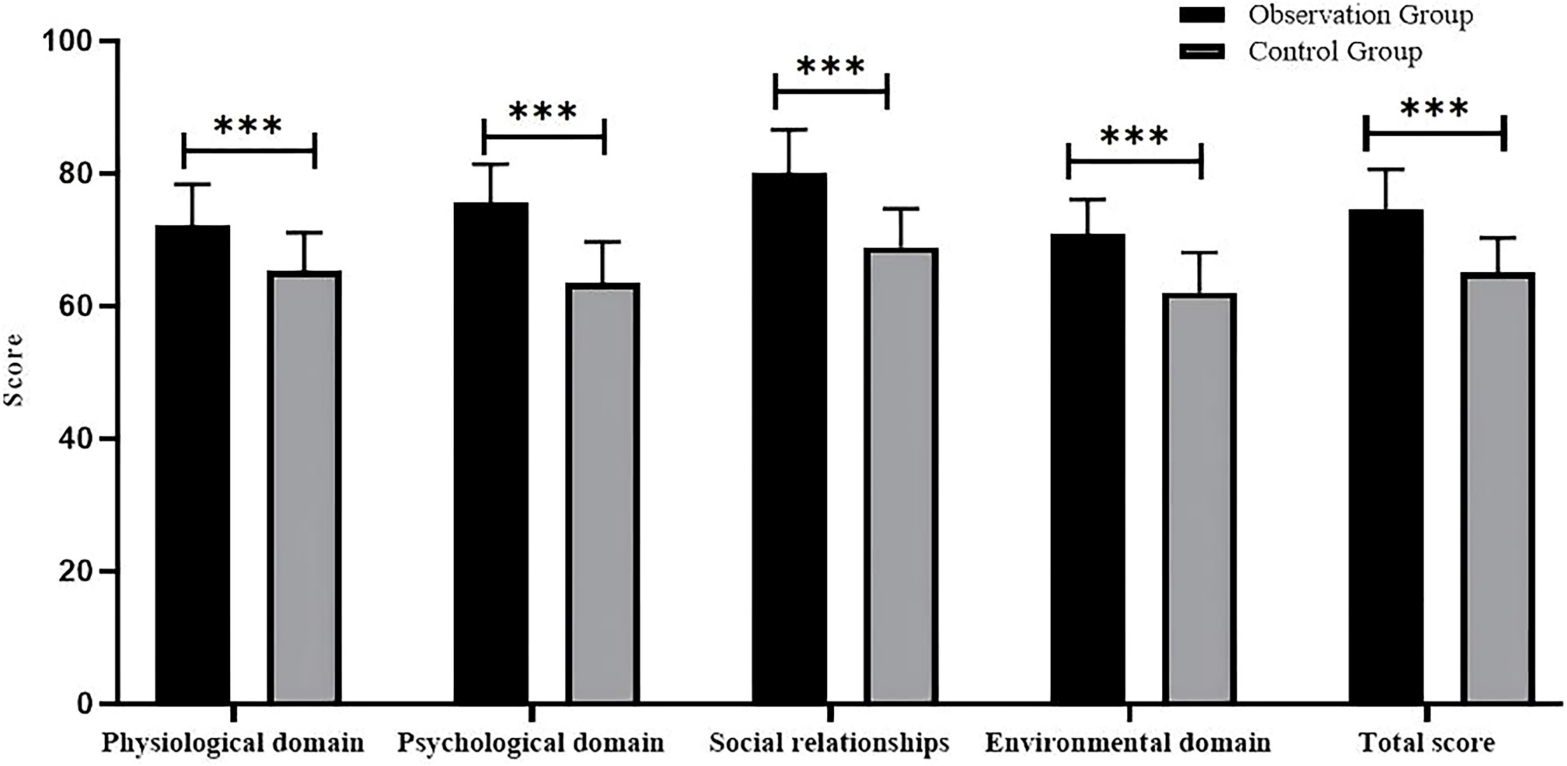
Comparison of WHOQOL-BREF scores across domains between the two groups after intervention. ***P < 0.001.

The observation group scored significantly higher than the control group on all dimensions of the WHOQOL-BREF scale (*P* < 0.05), with the most significant improvements in the psychological and social relations domains (Figure 2 and Table 3). This graph clearly reflects the score gap between the two groups in each domain, especially highlighting the apparent advantages of the observation group in psychological and social relationship dimensions.

**TABLE 3:** Comparison of quality of life scores between the two groups after intervention (x̄ ± s, points).

Group	Physiological domain	Psychological domain	Social relationships	Environmental domain	Total score
Observation group	72.3 ± 6.1*	75.6 ± 5.8*	80.2 ± 6.4*	70.8 ± 5.3*	74.7 ± 5.9*
Control group	65.4 ± 5.7	63.5 ± 6.2	68.9 ± 5.8	62.1 ± 6.0	65.0 ± 5.3
*T*	5.82	10.11	9.23	7.54	8.76
*P*	< 0.001	<0.001	< 0.001	<0.001	< 0.001

Compared with the control group,

*P < 0.05.

### Postoperative pain comparison

Postoperative throat pain levels were compared between the two groups (Table 4). The chi-square test showed a significant difference in pain distribution (χ^2^ = 13.19, df = 3, *P* = 0.004): 74.0% of patients in the observation group reported no pain or mild pain (Grade 0-I), while 62.0% of patients in the control group had moderate to severe pain (Grade II-IV), indicating lower pain levels in the observation group.

**TABLE 4:** Comparison of postoperative throat pain levels between the two groups.

Group	N	Grade 0 (n, %)	Grade I (n, %)	Grade II (n, %)	Grade III-IV (n, %)
Observation group	50	8(16.0%)	29(58.0%)	8(16.0%)	5(10.0%)
Control group	50	4(8.0%)	15(30.0%)	18(36.0%)	13(26.0%)

### Nursing satisfaction and complications

#### Nursing satisfaction

The satisfaction score in the observation group (92.4 ± 4.1) was significantly higher than that in the control group (80.3 ± 5.6) (*P* < 0.05).

#### Complications

The overall incidence rate in the observation group was 6.0% (3/50), including two cases of hoarseness and one case of limb convulsions; in the control group, it was 20.0% (10/50), including four cases of hoarseness, three cases of limb convulsions, and three cases of incision infection (*P* < 0.05).

## Discussion

This study, confirms that comprehensive nursing interventions combined with psychological care demonstrate significant advantages over routine care in the postoperative recovery of thyroid cancer patients. Patients in the observation group demonstrated superior improvements across multiple key indicators, including negative emotions, quality of life, postoperative pain control, nursing satisfaction, and complication rates (17). These findings align with trends observed in relevant domestic and international studies, providing robust evidence for optimizing postoperative care models for thyroid cancer patients (18).

First, regarding psychological and emotional improvement, the observation group exhibited significantly lower SAS and SDS scores post-intervention compared to the control group. This positive outcome primarily stems from the integration of multiple structured psychological support strategies within the intervention protocol. Mindfulness-based interventions effectively reduced catastrophic thinking about disease uncertainty and future recurrence by guiding patients to focus on the present moment and accept their emotions (19). Cognitive Behavioral Therapy (CBT) more specifically assisted patients with high anxiety/depression scores in identifying and restructuring automatic negative thoughts like “cancer will inevitably recur.” By increasing pleasurable experiences through behavioral activation, CBT disrupted the vicious cycle of emotions at both cognitive and behavioral levels (20, 21). Furthermore, progressive muscle relaxation training—specifically targeting neck and shoulder muscle groups (key areas affected by surgery)—effectively alleviated surgical site tension. This was reflected in our study results: 74.0% of patients in the observation group reported no or mild throat pain (Grade 0-I), compared to only 38.0% in the control group (Table 4). The reduction in physical tension further relieved anxiety-related somatic discomfort (e.g., muscle stiffness), which aligns with previous findings that relaxation training improves pain-related emotional distress (22). These structured psychological interventions (mindfulness, CBT, relaxation training) together addressed patients’ emotional and physical needs, forming a targeted support system.

Secondly, regarding quality of life improvement, the observation group demonstrated significantly higher scores than the control group across all four domains of the WHOQOL-BREF scale, with particularly notable enhancements in psychological and social relationships domains. This suggests that comprehensive nursing interventions prioritize not only patients’ physical recovery but also their overall psychosocial wellbeing. By establishing a tripartite team involving physicians, nurses, patients, and families, and convening family meetings, the patients’ social support systems were strengthened. This clarified family members’ roles in the rehabilitation process and enhanced patients’ sense of belonging and support within family relationships, directly reflected in improved scores in the social relations domain (23). Simultaneously, effective stress reduction and cultivation of positive emotions naturally form the core of enhancing psychological quality of life. In the observation group, WeChat-based transitional care (e.g., daily rehabilitation videos, weekly expert Q&A, real-time data collection via the Questionnaire Star mini-program) ensured continuity of care from hospital to home. This was associated with tangible improvements in our study: the observation group had a significantly higher nursing satisfaction score (92.4 ± 4.1 vs. 80.3 ± 5.6 in the control group) and a lower complication rate (6.0% vs. 20.0% in the control group). The dynamic adjustment of pain and sleep interventions based on real-time data likely enhanced patients’ adherence to rehabilitation, which in turn improved their quality of life—consistent with prior research showing digital health interventions improve post-cancer rehabilitation outcomes (24, 25).

Regarding postoperative pain management, patients in the observation group exhibited significantly less pharyngeal pain than the control group. Beyond conventional pain care, the comprehensive intervention employed in this study likely alleviated pain through multiple indirect pathways. Psychological interventions reduced anxiety levels, which has been demonstrated to lower pain thresholds and intensify pain perception (26). Relaxation training directly reduced muscle tension in the neck and throat, alleviating secondary pain caused by muscle spasms. This suggests that integrating psychological and relaxation techniques into postoperative care serves as an effective non-pharmacological adjunct to achieve multimodal analgesia and reduce reliance on pure medication.

Additionally, the observation group demonstrated significantly higher nursing satisfaction and a markedly reduced overall incidence of postoperative complications. This heightened satisfaction stems from the model’s core “patient-centered” philosophy, which comprehensively addresses patients’ informational, emotional, and support needs through personalized rehabilitation plans, continuous health education, convenient communication channels (WeChat platform), and in-depth psychological care. The reduction in complication rates may be attributed to more rigorous condition monitoring, standardized health guidance (e.g., neck function exercises, wound care), and improved treatment adherence resulting from enhanced patient emotional wellbeing and satisfaction.

This study has several limitations. First, as a retrospective study, it may be subject to selection bias. Second, the sample size is relatively small and derived from a single center, limiting the generalizability of findings, which requires further validation through multicenter, large-sample prospective randomized controlled trials. Third, the intervention period of 3 months necessitates longer-term follow-up to assess the long-term impact on negative emotions and quality of life.

## Conclusion

Comprehensive nursing interventions combined with psychological care can effectively alleviate postoperative negative emotions, improve quality of life, and enhance nursing satisfaction in thyroid cancer patients through multidimensional interventions addressing physiological, psychological, and social factors.

## Data Availability

The raw data supporting the conclusions of this article will be made available by the authors, without undue reservation.
